# The Brewed Rice Vinegar *Kurozu* Increases HSPA1A Expression and Ameliorates Cognitive Dysfunction in Aged P8 Mice

**DOI:** 10.1371/journal.pone.0150796

**Published:** 2016-03-04

**Authors:** Hiroaki Kanouchi, Toshiaki Kakimoto, Hideya Nakano, Masahiro Suzuki, Yuji Nakai, Kazuhiro Shiozaki, Kohei Akikoka, Konosuke Otomaru, Masanobu Nagano, Mitsuharu Matsumoto

**Affiliations:** 1 Department of Veterinary Pathobiology, Joint Faculty of Veterinary Medicine, Kagoshima University, Korimoto, Kagoshima, Japan; 2 Institute for Food Sciences, Hirosaki University, Yanagawa, Aomori, Japan; 3 Faculty of Fisheries, Kagoshima University, Shimoarata, Kagoshima, Japan; 4 Department of Veterinary Histopathology, Joint Faculty of Veterinary Medicine, Kagoshima University, Korimoto, Kagoshima, Japan; 5 Veterinary Clinical Center, Joint Faculty of Veterinary Medicine, Kagoshima University, Korimoto, Kagoshima, Japan; 6 Sakamoto Kurozu, Inc., Uenosono-cho, Kagoshima, Japan; 7 Department of Veterinary Anatomy, Joint Faculty of Veterinary Medicine, Kagoshima University, Korimoto, Kagoshima, Japan; Ehime University Graduate School of Medicine, JAPAN

## Abstract

*Kurozu* is a traditional Japanese rice vinegar. During fermentation and aging of the *Kurozu* liquid in an earthenware jar over 1 year, a solid residue called *Kurozu Moromi* is produced. In the present study, we evaluated whether concentrated *Kurozu* or *Kurozu Moromi* could ameliorate cognitive dysfunction in the senescence-accelerated P8 mouse. Senescence-accelerated P8 mice were fed 0.25% (w/w) concentrated *Kurozu* or 0.5% (w/w) *Kurozu Moromi* for 4 or 25 weeks. *Kurozu* suppressed cognitive dysfunction and amyloid accumulation in the brain, while *Kurozu Moromi* showed a tendency to ameliorate cognitive dysfunction, but the effect was not significant. We hypothesize that concentrated *Kurozu* has an antioxidant effect; however, the level of lipid peroxidation in the brain did not differ in senescence-accelerated P8 mice. DNA microarray analysis indicated that concentrated *Kurozu* increased HSPA1A mRNA expression, a protein that prevents protein misfolding and aggregation. The increase in HSPA1A expression by *Kurozu* was confirmed using quantitative real-time PCR and immunoblotting methods. The suppression of amyloid accumulation by concentrated *Kurozu* may be associated with HSPA1A induction. However, concentrated *Kurozu* could not increase HSPA1A expression in mouse primary neurons, suggesting it may not directly affect neurons.

## Introduction

Dementia is a common illness that affects the quality of life in the aging population. To date, there are no effective treatments; however, an early diagnosis and preventative measures, such as exercise, a healthy diet and social activity, have proven beneficial. Alzheimer’s disease (AD) and cerebrovascular disease frequently co-exist and are part of a syndrome that may result in dementia. Excess oxidative stress has been suggested to contribute to dementia progression [1, 2]. It has been reported that antioxidants from fruit, green tea or olive oil, which contain high concentrations of polyphenols, help to prevent cognitive dysfunction in animal studies [1, 2]. However, further evaluation is needed on potential candidates that ameliorate cognitive dysfunction.

In this study, we focused on the traditional Japanese black vinegar called *Kurozu*. *Kurozu* is made from steamed rice. Saccharification, alcoholization, and acetification from starch to acetic acid occurs in the same earthenware jar and the produced vinegar is left to age for over 1 year. The liquid in the jar is filtered to produce *Kurozu*, and the remaining extract is called *Kurozu Moromi* (*KM*). It has been reported that *Kurozu* and *KM* have several health benefits. *Kurozu* protects against colitis caused by dextran sulfate sodium [3], and suppresses proliferation of various cancer cell lines [4]. *Kurozu* also has an antioxidant effect [5]. A 10-fold concentrated form of *Kurozu* (*CK*) is prepared by vacuum distillation using a rotary evaporator. Using this process, acetic acid in *CK* is evaporated.

In the present study, we evaluated whether *CK* or *KM* could prevent cognitive dysfunction in senescence-accelerated P8 (P8) mice. The P8 mouse has been reported to be a good model for use in AD research [6–10]. The P8 mouse is one of nine senescence-prone strains of senescence-accelerated mice, which are originally generated from AKR/J mice. P8 mice exhibit a number of features that are known to occur in the pathogenesis of AD, such as increased oxidative stress, loss of neurons, gliosis, β amyloid alterations, and tau phosphorylation, as well as age-related deterioration in memory and learning. The senescence-resistance (R1) mouse is also generated from AKR/J mice at the same time. The R1 mouse shows normal aging and were used as the control mice in P8 mouse studies. The cognitive function of P8 mice following a diet of *CK* or *KM* was tested using the Morris water maze test.

Our aim was to identify a new candidate for the prevention of dementia, and we conclude that *CK* could ameliorate cognitive dysfunction.

## Materials and Methods

### Preparation of *CK* and *KM*

The *CK* diet included 0.25% (w/w) CK in CE-2 basic rodent diet (Nihon CLEA, Tokyo, Japan). *CK* was made from *Kurozu* liquid (Sakamoto Kurozu, Fukuyama, Kagoshima, Japan) by repeated vacuum distillation. The *KM* diet included 0.5% (w/w) *KM* powder in CE-2 diet. *KM* powder (Sakamoto Kurozu) was made from the squeezed residue following *Kurozu* production. The squeezed residue was dried under a vacuum at 110°C. The chemical composition of *CK* was 80% water, 9% crude protein (calculated as mineral nitrogen × 6.25), 2.5% organic acid, 5% ash, and 1% carbohydrate. The chemical composition of *KM* was 4% water, 12% crude protein (calculated as mineral nitrogen × 6.25), 23% organic acid, 1% ash, and 60% carbohydrate.

### Animal experiments

R1 and P8 mice were purchased from Japan SLC (Shizuoka, Japan). Mice were housed at 25±2°C with 55±10% humidity on a 12-h light/dark cycle (lighting time 08:00–20:00). All mice were housed in independent cages and had free access to food and water. This study was carried out in strict accordance with the recommendations in the guide for the humane treatment and management of animals of the Japanese Law (No. 105) and Notification (No. 6). The protocol was approved by the Committee on the Ethics of Animal Experiments of the Kagoshima University Committee for Animal Experiment (Permit Number: A10030 and VM12018). All mice were killed by bleeding under isoflurane anesthesia, and all efforts were made to minimize suffering.

### Experiment 1

Ten-week-old male R1 mice (n = 16) were fed a control CE2 diet. P8 mice were divided into three groups as follows: control CE2 diet group (n = 9); *KM* diet group (n = 9); and *CK* diet group (n = 9). Feeding of experimental diets started from 12 weeks of age until the mice were killed. The water maze test began when mice were 15 weeks of age and continued for 16 days. All mice were killed under anesthesia at 17 weeks old (4 months old). Serum was collected to measure the levels of thiobarbituric acid reactive substances (TBARS), alanine aminotransferase (ALT), and aspartate aminotransferase (AST) using a commercial kit (Cayman Chemical, Ann Arbor, MI; Dry-chem Chemistry analyzer, Tokyo, Japan). Three mice from each group were fixed with neutralized 10% (v/v) formalin by perfusion fixation to obtain the brain. Four-μm-thick tissue sections were prepared from paraffin-embedded brains and were used for the detection of aggregated protein using the ProteoStat Amyloid Plaque Detection Kit (Enzo Life Sciences Inc., Farmingdale, NY) according to the manufacturer’s instructions. The detection reagent interacts with the cross-β-sheet quaternary structure of amyloid fibrils on the slides and is readily excited by an argon ion laser source, with an emission maximum of 600 nm. Fluorescent intensities in the cerebral cortex and hippocampus were detected using a confocal laser microscope (EZ-C1, Nikon, Tokyo, Japan). Brain homogenates were prepared to measure protein concentration (Pierce BCA Protein Assay Kit, Pierce, Rockford, IL) and TBARS. TBARS levels are expressed as MDA concentration. Heat shock 70 kDa protein 1A (HSPA1A) in brain homogenates was evaluated using the Hsp70 High-Sensitivity ELISA Kit (StressMarq Biosciences, British Columbia, Canada) according to the manufacturer’s instructions.

### Experiment 2

Twelve 4-week-old male R1 mice and 36 4-week-old male P8 mice were purchased from Japan SLC. Mice were housed under normal conditions until 11 weeks of age, after which experimental diets began. R1 mice were fed a control CE2 diet. P8 mice were divided into three groups as follows: control CE2 diet group (n = 12); *KM* diet group (n = 12); and *CK* diet group (n = 12). After 24 weeks (8.4 months old), the water maze test was started.

### Morris water maze test

The standard Morris water maze test was used with minor modifications [11]. A circular pool (100 cm in diameter) was filled with water (17-cm-deep, 25°C) and divided into four quadrant zones: east, west, south, and north. A clear platform (10 cm in diameter and 16 cm in height) was hidden in the center of the right upper quadrant, submerged 1 cm below the water’s surface. Mice were trained to find the hidden platform in the water maze for 15 (Experiment 1) or 4 (Experiment 2) consecutive days, three trials per day. Mice were not allowed to search for the platform for more than 60 s, after which they were guided to the platform and allowed to stay on the platform for 15 s. On the last day, a probe trial test was performed for a period of 120 s without the platform. In each trial, the swimming path and escape time for locating the hidden platform was recorded using a web camera (Logicool HD Webcam C270, Logicool, Lausanne, Switzerland) and analyzed using a tracking system (TopScan Lite 2.0, Clever Sys, Reston, VA).

### Antioxidant activities

The 2,2-diphenyl-1-picrylhydrazyl (DPPH) radical scavenging assay, superoxide radical scavenging assay, and ferric-reducing antioxidant power assay were measured according to Ikeda *et al*. [12]. Briefly, 25 μL *CK* was used as a test sample in 1 mL of reaction mix in all assays. The hydroxyl radical scavenging activity was measured as follows: 25 μL *CK* was mixed with 25 μL of 9.1 mM salicylic acid, 25 μL of 9.1 mM ferrous sulfate, and 500 μL of 8.8 mM hydrogen peroxide. The reaction mixture was incubated for 10 min at room temperature, after which the absorbance of the mixture was measured at 510 nm using a UV/Vis spectrophotometer (Shimadzu, Tokyo, Japan). The percentage of hydroxyl radical scavenging activity of the test sample was determined in comparison with the negative control. Various concentrations of reduced ascorbic acid were used as positive controls. The negative control contained neither ascorbic acid nor *CK*.

### DNA microarray analysis and quantitative real-time PCR

The left side of the hippocampus region was excised from the brains of four mice in each group. Total RNA was extracted using the RNeasy Mini Kit (Qiagen, Valencia, CA). RNA quantity, purity, and concentration were determined using a nanodrop (Amersham Biosciences, Foster City, CA) and Experion RNA StdSens (BioRad Laboratories, Hercules, CA). Total RNA was used for microarray analysis and quantitative real-time PCR, as described below. DNA microarray analysis was performed according to the manufacturer’s instructions. We used GeneChip Mouse Gene 1.0 ST Array (Affymetrix, Santa Clara, CA). Fluorescent signals were scanned using the Affymetrix GeneChip System. Data analysis of the DNA microarray was carried out as described previously [13], except for normalization methods. Briefly, CEL files were normalized using the robust multi-array average method [14]. Hierarchical clustering was then performed using the pvclust() function in R. To identify differentially expressed genes, the rank products method [15] was applied to robust multi-array average normalized data. Probe sets with a false discovery rate <0.05 were regarded as having different expression levels between the two groups (R1 CE2 *vs*. P8 CE2, or P8 CE2 *vs*. *CK*). The annotation file for the Mouse Gene 1.0 ST Array was downloaded from the Affymetrix website. The data discussed in this publication have been deposited in NCBI’s Gene Expression Omnibus [16] and are accessible through GEO series accession number GSE70514 (http://www.ncbi.nlm.nih.gov/geo/query/acc.cgi?acc=GSE70514). Quantitative real-time PCR analyses were performed using a Lightcycler 1.5 (Roche Diagnostics, Tokyo, Japan) and SYBR Premix Ex Taq (Takara Bio, Shiga, Japan). The primer sets for HSPA1A and glyceraldehyde 3-phosphate dehydrogenase (GAPD) were purchased from Takara Bio. Data are presented as relative values (HSPA1A/GAPD).

### Mouse primary neuronal cultures

Pregnant C57BL/6N mice were killed by bleeding under isoflurane anesthesia. Brains were excised from embryonic (E18) mice. Neurons were collected from the cerebrum and hippocampus using Nerve-Cell Culture System/Dissociation Solutions (Sumitomo Bakelite, Tokyo, Japan). Neurons (2.5 × 105 cells/well) were plated on a 24-well plate (Celltight PL plate, Sumitomo) and cultured in Neuron Assay Medium (Sumitomo). After 4 days, media was replaced with fresh media containing various concentrations of *CK* (0, 0.00125, 0.0025, 0.005, or 0.01% (v/v)). After 24 h, total RNA was extracted from neurons with TRIzol (Invitrogen Life Technologies, Carlsbad, CA). Quantification of HSPA1A mRNA was carried out as described above.

### Statistical analysis

Data were analyzed by one-way ANOVA with Tukey’s post-hoc analysis (Statistical Package for the Social Sciences, v.17; SPSS Inc., Chicago, IL). A p-value <0.05 was considered statistically significant. All data are presented as mean ± SE. In Experiment 2, one mouse in the P8 *CK* group was euthanized because it was showing signs of severe dermatitis. All data from this mouse were removed from the study.

## Results

### Effects of *KM* or *CK* feeding on cognitive function in P8 mice

#### Experiment 1

**Fig 1:**
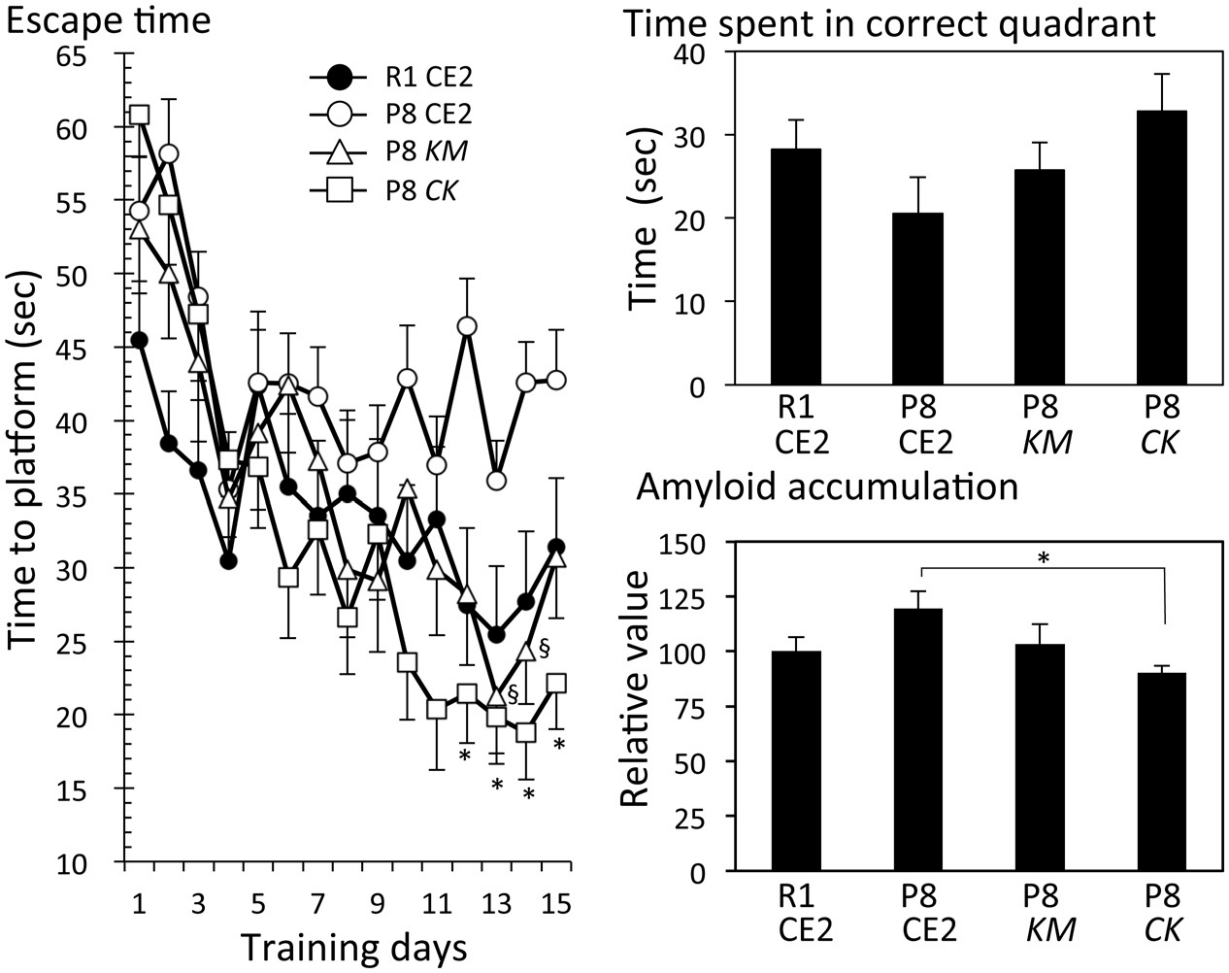
Evaluation of cognitive function and amyloid accumulation in 4-month-old senescence-accelerated mice. P8 mice were fed a normal diet (P8, n = 9), *Kurozu Moromi* containing diet (*KM*, n = 9), or a concentrated *Kurozu* containing diet (*CK*, n = 9) for 5 weeks from 12 weeks old. R1 mice were fed a normal diet (R1, n = 16). Cognitive function was evaluated using the Morris water maze test at 15 weeks of age. Left panel shows escape time during the training phase (R1 CE2, n = 16; P8 CE2, n = 9; P8 *KM*, n = 9; P8 *CK*, n = 9). ^§^p<0.05, P8 CE2 *vs*. P8 *KM*; *p<0.05, P8 CE2 *vs*. P8 *CK*. Right upper panel shows results of the probe test. Right lower panel shows amyloid accumulation (R1 CE2, n = 3; P8 CE2, n = 3; P8 *KM*, n = 3; P8 *CK*, n = 3). Results are expressed as mean ± SE. *p<0.05, *vs*. P8 CE2.

In Experiment 1, mice were 15–17 weeks old at the time the Morris water maze test was performed. These mice were fed *KM* or *CK* for 5 weeks. There were no significant differences in body weight gain and serum ALT and AST levels among the groups (S1 Table). The Morris water maze test showed that the escape time of the R1 CE2 group gradually shortened compared with that of the P8 CE2 group during training days, but there were no significant differences (Fig 1, left panel). Escape times for the P8 *KM* and P8 *CK* groups were faster than that of the P8 CE2 group. Significant differences were observed at 12 to 15 days in the P8 *CK* group compared with the P8 CE2 group, and 13 to 14 days in the P8 *KM* group compared with the P8 CE2 group.

Following the completion of the Morris water maze test (day 15), the probe test was performed without the platform to evaluate working memory (Fig 1, upper right panel). As a result, the time spent in the correct quadrant in the P8 *CK* group was slightly longer than that of the P8 CE2 group (p = 0.11). The time spent by the P8 *KM* group was also slightly longer, but there were no significant differences when compared with the P8 CE2 group. During the probe test, mice did not stop swimming, therefore, there were no differences in swim distance among all groups.

**Fig 2:**
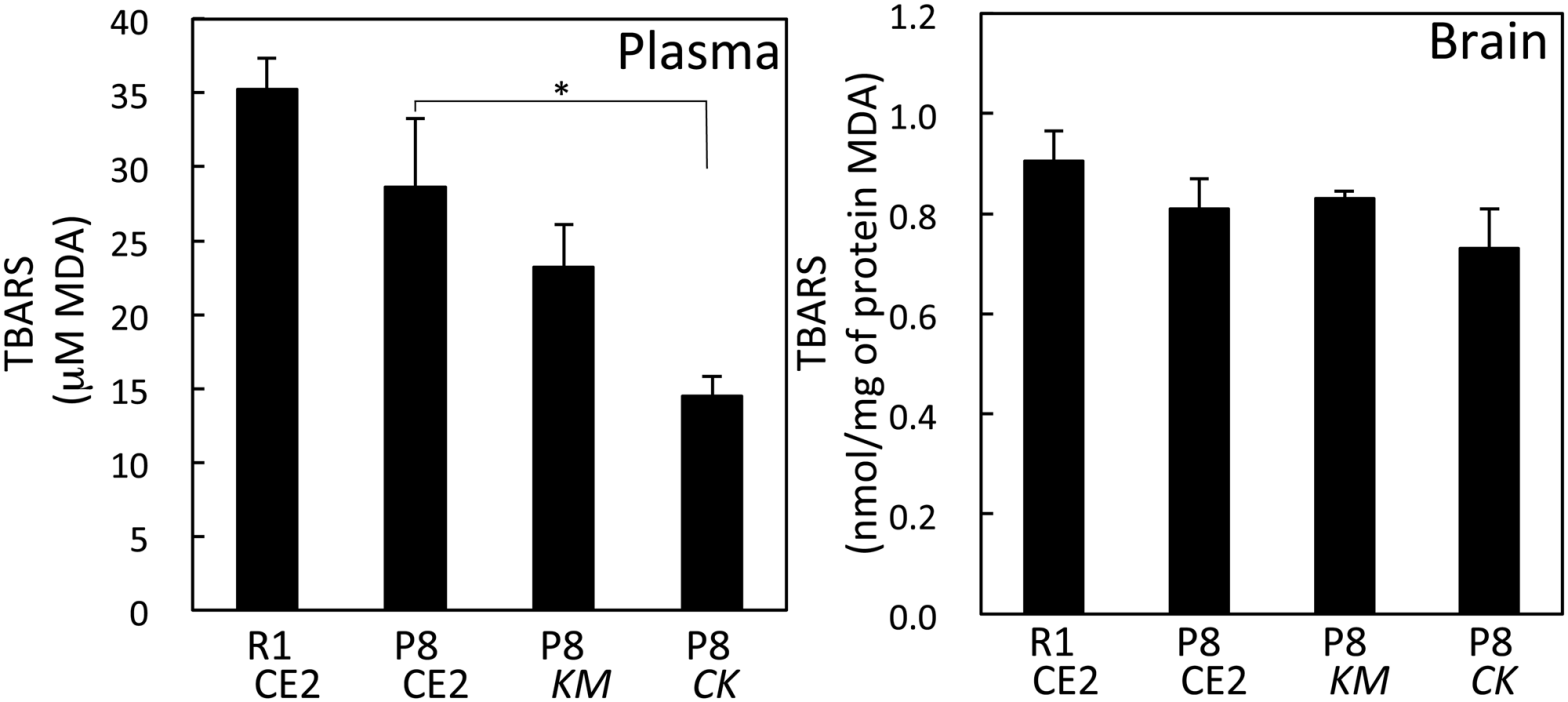
Level of TBARS in serum and brain homogenates in 4-month-old senescence-accelerated mice. Levels of TBARS in serum were measured (R1 CE2, n = 16; P8 CE2, n = 9; P8 *KM*, n = 9; P8 *CK*, n = 9) and brain homogenates (R1 CE2, n = 13; P8 CE2, n = 6; P8 *KM*, n = 6; P8 *CK*, n = 6). Results are expressed as mean ± SE. *p<0.05, *vs*. P8.

We attempted to detect neuritic plaques in brain sections using a fluorescent dye that binds to aggregated protein, but no clear neuritic plaques could be detected for any group. This result could be because of the young age of the mice. However, the fluorescence intensity of aggregated protein in the cerebral cortex and hippocampus was significantly lower in the P8 *CK* group compared with the P8 CE2 group (p<0.05). In serum, the MDA value in the P8 *CK* group was significantly lower than that of the P8 CE2 group. However, in brain homogenates, the MDA values were not different among groups (Fig 2).

#### Experiment 2

**Fig 3:**
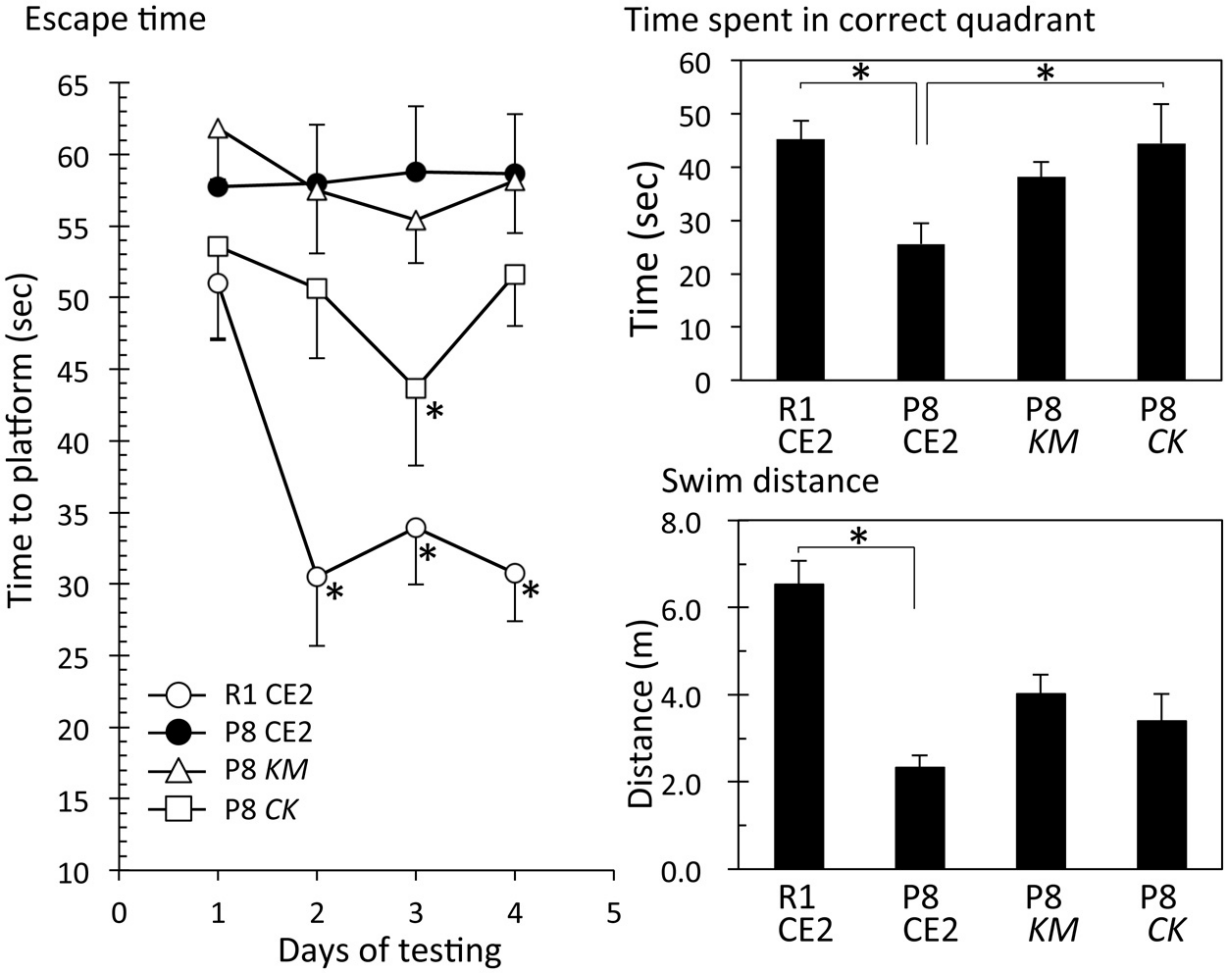
Evaluation of cognitive function in 8.4-month-old senescence-accelerated mice. P8 mice were fed a normal diet (P8 CE2, n = 12), *Kurozu Moromi* containing diet (*KM*, n = 12), or concentrated *Kurozu* containing diet (*CK*, n = 12) for 24 weeks from 12 weeks old. Cognitive function was evaluated using the Morris water maze test at 35 weeks of age. Left panel shows escape time during the training phase (R1 CE2, n = 12; P8 CE2, n = 12; P8 *KM*, n = 12; P8 *CK*, n = 11). Right upper panel shows the results of the probe test (R1 CE2, n = 12; P8 CE2, n = 12; P8 *KM*, n = 12; P8 *CK*, n = 11). Right lower panel shows swimming distance during the probe test (R1 CE2, n = 12; P8 CE2, n = 12; P8 *KM*, n = 12; P8 *CK*, n = 11). Results are expressed as mean ± SE. *p<0.05, *vs*. P8.

In Experiment 2, mice were 7 months old at the time the Morris water maze test was performed. These mice were fed KM or *CK* for 4 months. Body weight gains for P8 CE2, P8 *KM*, and P8 *CK* groups were significantly lower than that of the R1 group. *KM* or *CK* feeding did not affect the body weight of P8 mice. The difference observed in body weights between SAM R1 and SAM P8 is thought to be the result of aging. Serum ALT levels were not different among the groups (S2 Table). Escape time in the Morris water maze test revealed that the R1 group recognized the position of the platform, but that the P8 CE2 group and P8 *KM* did not (Fig 3, left).

**Fig 4:**
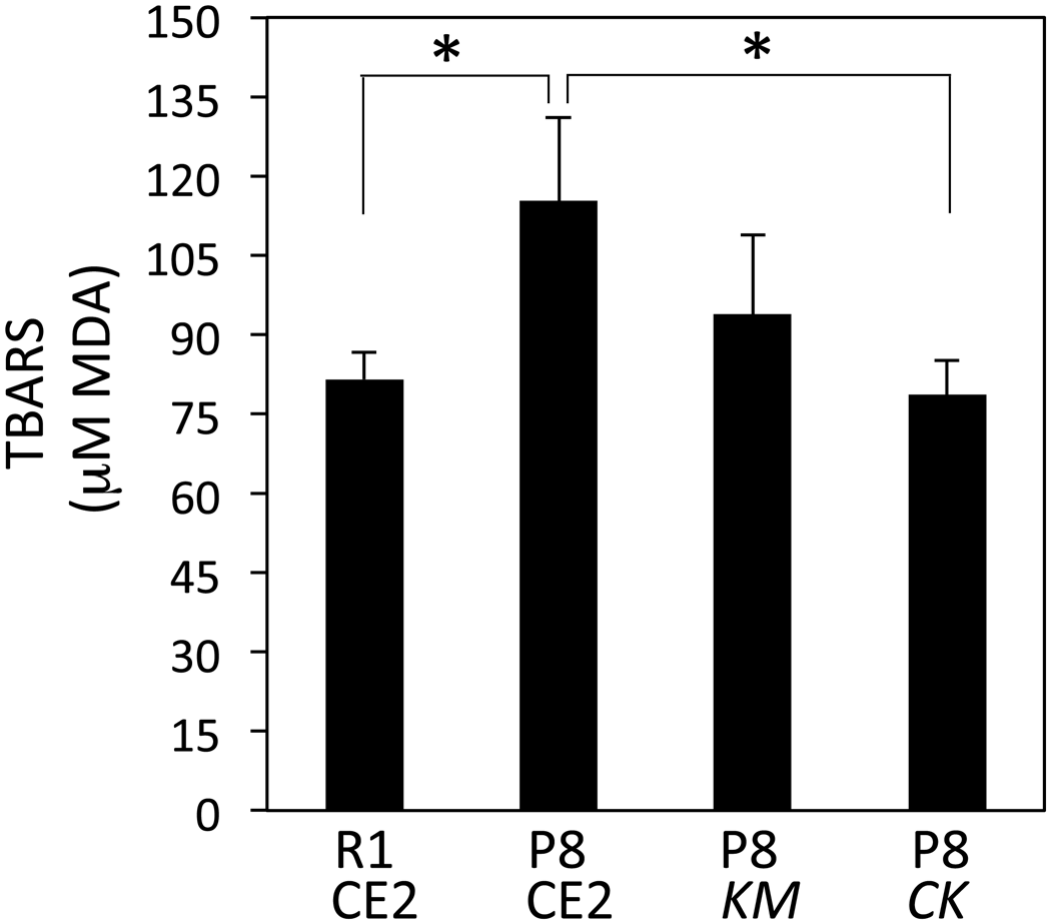
Level of TBARS in serum in 7-month-old senescence-accelerated mice. TBARS levels in serum were measured (R1 CE2, n = 12; P8 CE2, n = 12; P8 *KM*, n = 12; P8 *CK*, n = 11). Results are expressed as mean ± SE. *p<0.05, *vs*. P8 CE2.

In the Morris water maze test performed without the platform to evaluate working memory, the time spent in the correct quadrant was significantly shorter in the P8 CE2 group compared with the R1 CE2 group (Fig 3, upper right). The time spent for the P8 *CK* group, but not the P8 *KM* group, was significantly longer than that of the P8 CE2 group. Swim distance was significantly shorter for the P8 CE2 group compared with the R1 CE2 group. This result is thought to be a result of aging. As mice did not stop swimming during the probe test, the swim speed of P8 mice was slower than that of R1 mice. *KM* or *CK* feeding did not affect the swim distance in P8 mice (Fig 3, lower right). Serum MDA values of P8 CE2 mice were significantly higher compared with the R1 group (Fig 4). Values of P8 *CK* mice were significantly lower compared with P8 CE2 mice.

### Antioxidant activity of *CK*

The antioxidant activity of *CK* was evaluated using ferric reducing antioxidant assay, DPPH radical scavenging assay, superoxide radical scavenging assay, and hydroxyl radical scavenging assay (Table 1). Solidified *CK*, prepared by vacuum distillation, was dissolved in water. In the present study, to evaluate antioxidant activities under the same conditions, the activities were compared with ascorbic acid. These experiments showed that *CK* had a free radical scavenging activity against superoxide and hydroxyl radicals. These activities were equivalent to 1.03–5.50 mM of ascorbic acid.

**Table 1:** Antioxidant Activities of *CK*.

	2.5% *CK* in working solution	Equivalent to ascorbic acid (undiluted *CK*)
**Ferric reducing antioxidant power**	0.67±1.2 (OD_750_)	1.47±0.10 mM
**DPPH radical scavenging activity**	63.7±1.2%	5.50±0.13 mM
**Superoxide radical scavenging activity**	22.4± 2.4%	1.03±0.26 mM
**Hydroxyl radical scavenging activity**	62.3±0.9%	1.11±0.03 mM

Data are presented as the mean ± SE (n = 3).

### Effect of *CK* on gene expression in the brain-DNA microarray analysis

To identify changes in gene expression following *CK* feeding, DNA microarray analysis was carried out using cDNA libraries that were synthesized from total RNA of the R1 CE2 group, P8 CE2 group, and P8 *CK* group. Differentially expressed genes were identified using the rank products method [15]. If the false discovery rate was <0.05, the gene was regarded as significantly down- or up-regulated. As a result, 116 and 191 genes were down-regulated and up-regulated, respectively, in the P8 CE2 group compared with the R1 CE2 group (S3 and S4 Tables). Unfortunately, there were no genes related to AD or biological pathways known to improve cognitive function using the DAVID Bioinformatics Resources 6.7 (NIAID/NIH, Frederick, MD). Compared with the P8 CE2 group, 28 genes were up-regulated in the P8 *CK* group, and six genes were down-regulated in the P8 CE2 group compared with the R1 CE2 group (Table 2, left column). Compared with the P8 CE2 group, 39 genes were down-regulated in the *CK* group, and 21 genes were up-regulated in the P8 CE2 group compared with the R1 CE2 group (Table 2, right column).

**Table 2:** Genes Related to *CK* Feeding.

Up regulation						Down regulation					
Rank	RefSeq number	Symbol	Rank	RefSeq number	Symbol	Rank	RefSeq number	Symbol	Rank	RefSeq number	Symbol
**1**	**NM_001045550**	**Mup2**	21	NM_025586	Rpl15	1	**NM_019647**	**Rpl21**	21	**NM_021791**	**Doc2g**
2	NM_013721	Rpl7a	22	NM_009630	Adora2a	2	**NM_011312**	**S100a5**	22	NM_001289497	Cpa6
**3**	**NM_001110129**	**Ppih**	23	NM_026468	Atp5g2	3	**NM_001099641**	**Gabra6**	23	NM_178213	Hist2h2ab
**4**	**NM_010410**	**Hcrt**	24	NM_001146299	Sh3rf2	4	**NM_001126045**	**Smok3a**	24	NM_001097979	Hist1h2bq
5	NM_001048179	Ccl27a	**25**	**NM_001122647**	**Mup10**	5	NM_008252	Hmgb2	25	**NM_175651**	**Cnpy1**
6	NM_019647	Rpl21	26	NM_001001559	Usp17ld	6	**NM_001190332**	**Frmd7**	26	**NM_001034898**	**Ms4a15**
**7**	**NM_009654**	**Alb**	27	NM_146471	Olfr1393	7	**NM_130878**	**Cdhr1**	27	NM_133192	Npffr2
8	NM_010479	Hspa1a	28	NM_010118	Egr2	8	**NM_145399**	**Scgn**	28	NM_212457	Bex4
9	NM_007392	Acta2				9	NM_019820	Cbln3	29	NM_001291280	Nmb
10	NM_011561	Tdg				10	**NM_001080811**	**Bpifa6**	30	NM_013721	Rpl7a
11	NM_010478	Hspa1b				11	**NM_029726**	**Trdn**	31	**NM_177084**	**Slc9a4**
12	NM_174875	Atg4a				12	**NM_001164789**	**Eomes**	32	NM_024266	Rps25
13	NM_153553	Npas4				13	**NM_001013765**	**Zscan4c**	33	NM_001011847	Olfr591
14	NM_001276684	Arc				14	NM_011153	Ppp1r17	34	**NM_010551**	**Il16**
**15**	**NM_013795**	**Atp5l**				15	**NM_007592**	**Car8**	35	**NM_010894**	**Neurod1**
16	NM_022427	Gpr88				16	**NM_001145452**	**Arhgef33**	36	NM_010439	Hmgb1
17	NM_011455	Serpinb9g				17	NM_018730	Rpl36	37	NM_024266	Rps25
18	NM_181543	Gpr151				18	**NM_001029988**	**Fat2**	38	NM_025919	Rpl11
19	NM_025404	Arl4d				19	NM_001166741	Vmn1r121	39	NM_001045539	Xlr5a
20	NR_027799	Tbrg3				20	**NM_008117**	**Gh**			

Bolded genes showed opposite expression pattern in P8 CE2 compared with R1 CE2.

### HSPA1A expression

**Fig 5:**
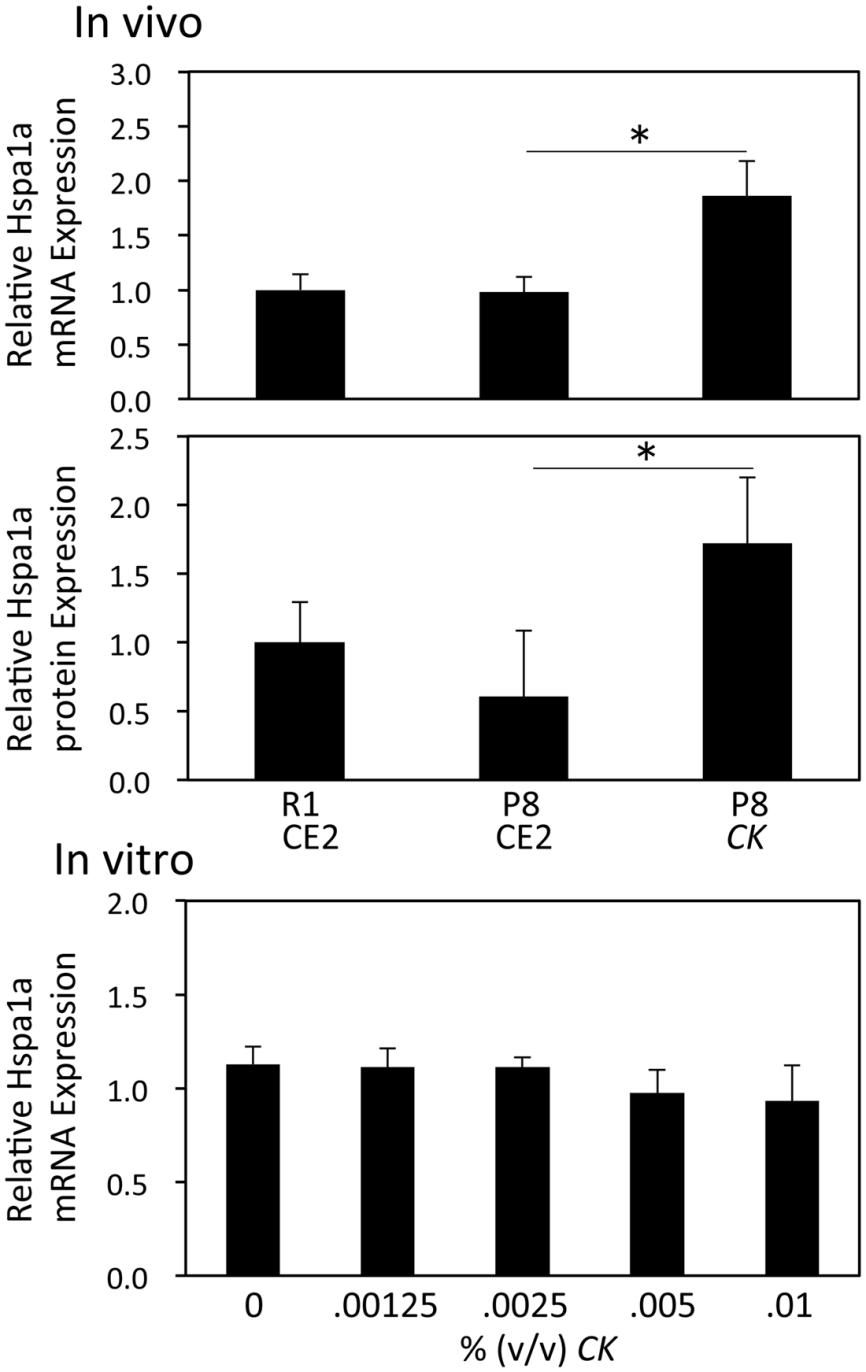
Effect of *CK* on the expression of HSPA1A mRNA and protein. Total RNA was prepared from brains of R1 CE2, P8 CE2, and P8 *CK* mice from Experiment 1 (n = 4). Expression levels of HSPA1A and GAPD mRNA were evaluated using quantitative real-time PCR. Results are presented as the relative HSPA1A mRNA level (HSPA1A/GAPD). HSPA1A protein expression in brain homogenates was evaluated using a commercial ELISA kit (R1 CE2, n = 11; P8 CE2, n = 4; P8 *CK*, n = 4). Total RNA was prepared from mouse primary neurons treated with *CK* for 24 h (n = 4). Expression levels of HSPA1A and GAPD mRNA were evaluated using quantitative real-time PCR. Results are presented as the relative HSPA1A mRNA level (HSPA1A/GAPD). Results are expressed as mean ± SE. *p<0.05, *vs*. P8 CE2.

*CK* decreased the amount of aggregated protein in the brain of P8 mice. DNA microarray analysis showed that HSPA1A, a protein that aids in folding of misfolded proteins, was up-regulated by *CK*. HSPA1A mRNA levels and protein expression were confirmed using real-time PCR and ELISA, respectively. Fig 5 shows that both mRNA and protein levels of HSPA1A significantly increased in the brains of *CK*-fed mice (p<0.05). However, exposure of primary neuronal cultures to *CK* for 24 h did not affect HSPA1A mRNA expression (Fig 5).

## Discussion

In this study, we examined whether *KM* or *CK* could protect against cognitive dysfunction in P8 mice. In Experiment 1, escape times in the Morris water maze test for the *KM* and *CK* groups were faster than that for the P8 CE2 group at 4 months of age. In the probe trial test on the last day, there were no significant differences in time spent in the correct quadrant among the P8 CE2, P8 *KM*, and P8 *CK* groups.

Mice fed a *CK* and *KM* diet did show a slight amelioration in cognitive dysfunction. Therefore, in Experiment 2, we evaluated the cognitive function of P8 mice at 7 months of age. Body weights of 7-month-old P8 mice decreased compared with R1 mice of the same age. The total swim distance of the P8 CE2 group in the Morris water maze test was significantly shorter compared with the R1 CE2 group, suggesting that the exercise activity of 7-month-old P8 mice declined. Escape time decreased after each training day in the R1 CE2 group. In contrast, there was no change in the P8 CE2 group. The time spent in the correct quadrant for the P8 CE2 group was also significantly shorter than that of the R1 CE2 group. These results indicate that 7-month-old P8 mice lost not only exercise activity but also cognitive ability. Escape time of the P8 *CK* group, but not the *KM* group, was slightly faster than that of the P8 CE2 group. There was a significant difference at day 3 (p<0.05). Time spent in the correct quadrant was significantly longer in *CK*-fed mice, but swimming distance did not change. These findings suggest that *CK* feeding ameliorates cognitive dysfunction, but not exercise ability, in older P8 mice.

Although we attempted to identify neuritic plaques and neurofibrillary tangles in the P8 CE2 mouse brain, none could be detected. However, aggregated protein increased in the P8 CE2 group and decreased in the P8 *CK* group. A previous report suggests that *CK* has a scavenging effect on DPPH and a suppressive effect on low-density lipoprotein oxidation [5]. Our *in vitro* experiments also showed that *CK* had antioxidant properties. Although we expected that *CK* would suppress oxidation, we could not sufficiently evaluate oxidation levels in the brain. Effective molecules from food are generally modified in the digestive tract, and these modified molecules may have different effects from the original molecules. Further experiments are required to determine whether *CK* feeding has effects on excessive oxidation in the brain.

*CK* ameliorated both cognitive dysfunction and the accumulation of aggregated protein in the brain more effectively than *KM*. We therefore focused on the effect of *CK* on cognitive dysfunction. To further understand the mechanism behind the effect of *CK*, we measured the change in gene expression in brains from R1 CE2, P8 CE2, and P8 *CK* groups. DNA microarray analysis showed that expression levels of many genes were altered in 4-month-old P8 mice compared with R1 mice of the same age. Unfortunately, we could not find genes directly related to brain function. Interestingly, changes in gene expression accompanied by aging were reversed in the P8 *CK* group. Six of the genes down-regulated in the P8 CE2 group were up-regulated in the P8 *CK* group, and 15 up-regulated genes in P8 CE2 mice were down-regulated in the P8 *CK* group. Moreover, *CK* feeding increased major urinary protein-2 (α2u-globulin), which dramatically decreases in the liver during aging [17]. However, its function in the brain has not been elucidated. Overall, *CK* ameliorates senescing of P8 mice.

It has been suggested that accumulation of tau and/or amyloid β oligomers in the brain initiates a cascade of pathological events resulting in neurodegeneration and cognitive decline [18]. Heat shock proteins (HSP) are known chaperones and play a major role in preventing protein misfolding and aggregation. HSPs may also prevent aggregation and oligomerization of amyloid β and tau protein [19]. Leak reviewed the relationship between HSPA1A expression and AD [20]. He concluded that although there is generally an increase in HSPA1A expression seen with mild cognitive decline, some authors have observed age-related reduction in HSP70 in studies using older male mice. Therefore, it is difficult to form conclusions about the expression level in AD. Bobkova *et al*. revealed that exogenous HSP70 inhibited the accumulation of amyloid β and cognitive abnormalities in a mouse model of AD [21]. DNA microarray analysis indicated that HSPA1A (also known as HSP70) was up-regulated by *CK*. This finding was confirmed using quantitative real-time PCR and ELISA. The decreased accumulation of aggregated protein in the P8 *CK* group may be related to the activation of HSPA1A. *In vitro* experiments using primary neurons revealed that the addition of *CK* to culture medium had no effect on HSPA1A mRNA expression levels. This result suggests that *CK* does not have a direct effect on neurons. Further studies are needed to clarify the induction mechanism of HSPA1A by *CK*.

## Conclusion

We evaluated the effect of *KM* or *CK* feeding on cognitive function in P8 mice. *CK* feeding ameliorated cognitive dysfunction and suppressed the accumulation of aggregated protein in the brain. *CK* increased HSPA1A expression in the brain. The activation of HSPA1A may be associated with the attenuated accumulation of aggregated protein in the brain.

## Supporting Information

S1 Table Body Weight and Serum ALT and AST Level at 4 Months Old. (TIF)

S2 Table Body Weight Gain and Serum ALT at 8 Months Old. (TIF)

S3 Table Down-regulated Genes in P8 Mice. (TIF)

S4 Table Up-regulated Genes in P8 Mice. (TIF)
