# Supplementary figures and images for: The Brewed Rice Vinegar Kurozu Increases HSPA1A Expression and Ameliorates Cognitive Dysfunction in Aged P8 Mice
Source: PLoS One. 2016 Mar 4;11(3):e0150796. doi: 10.1371/journal.pone.0150796 (PMC4778952; doi:10.1371/journal.pone.0150796)

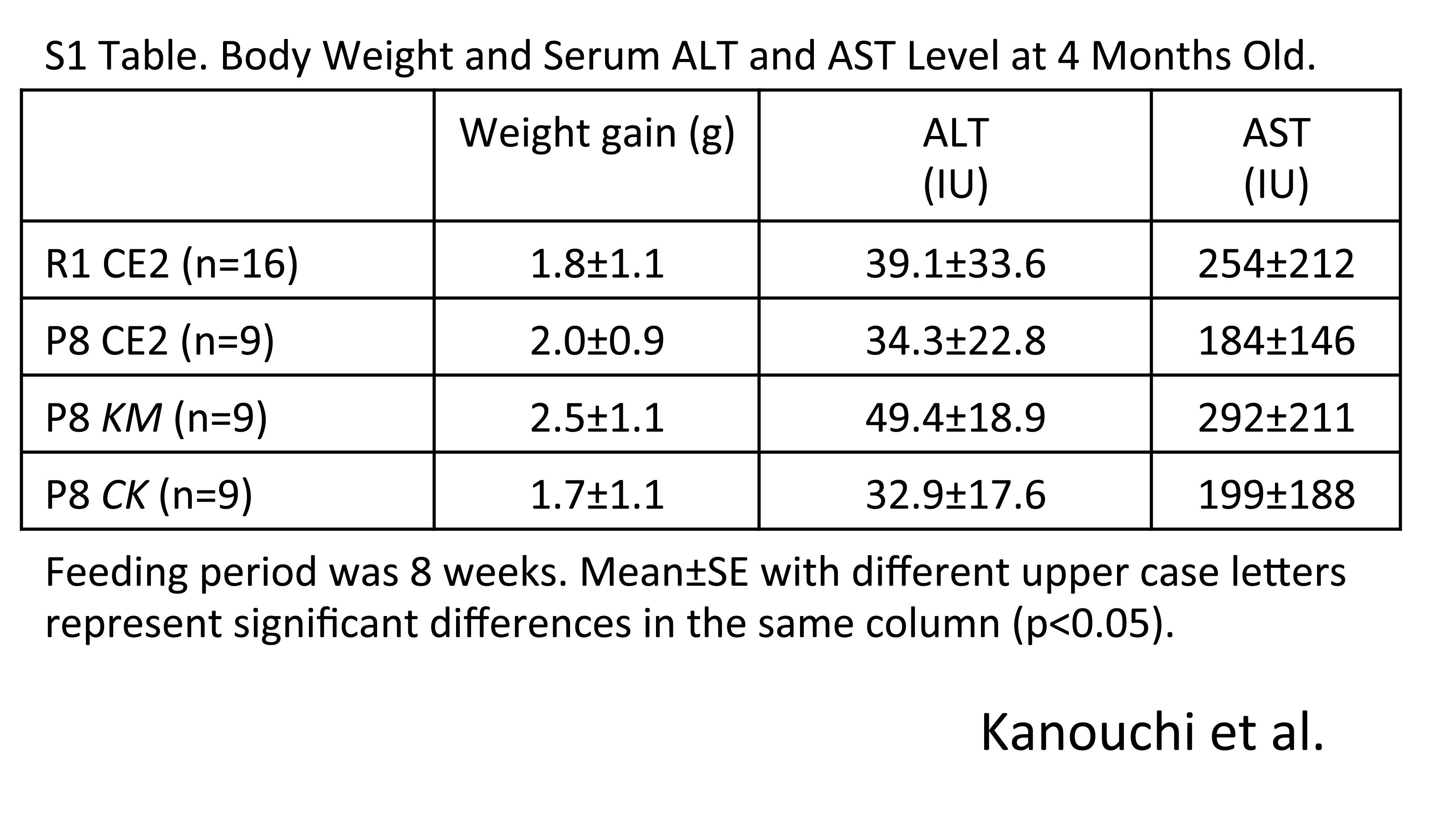

Supplement: S1 Table — (TIF) [file pone.0150796.s001.tif]

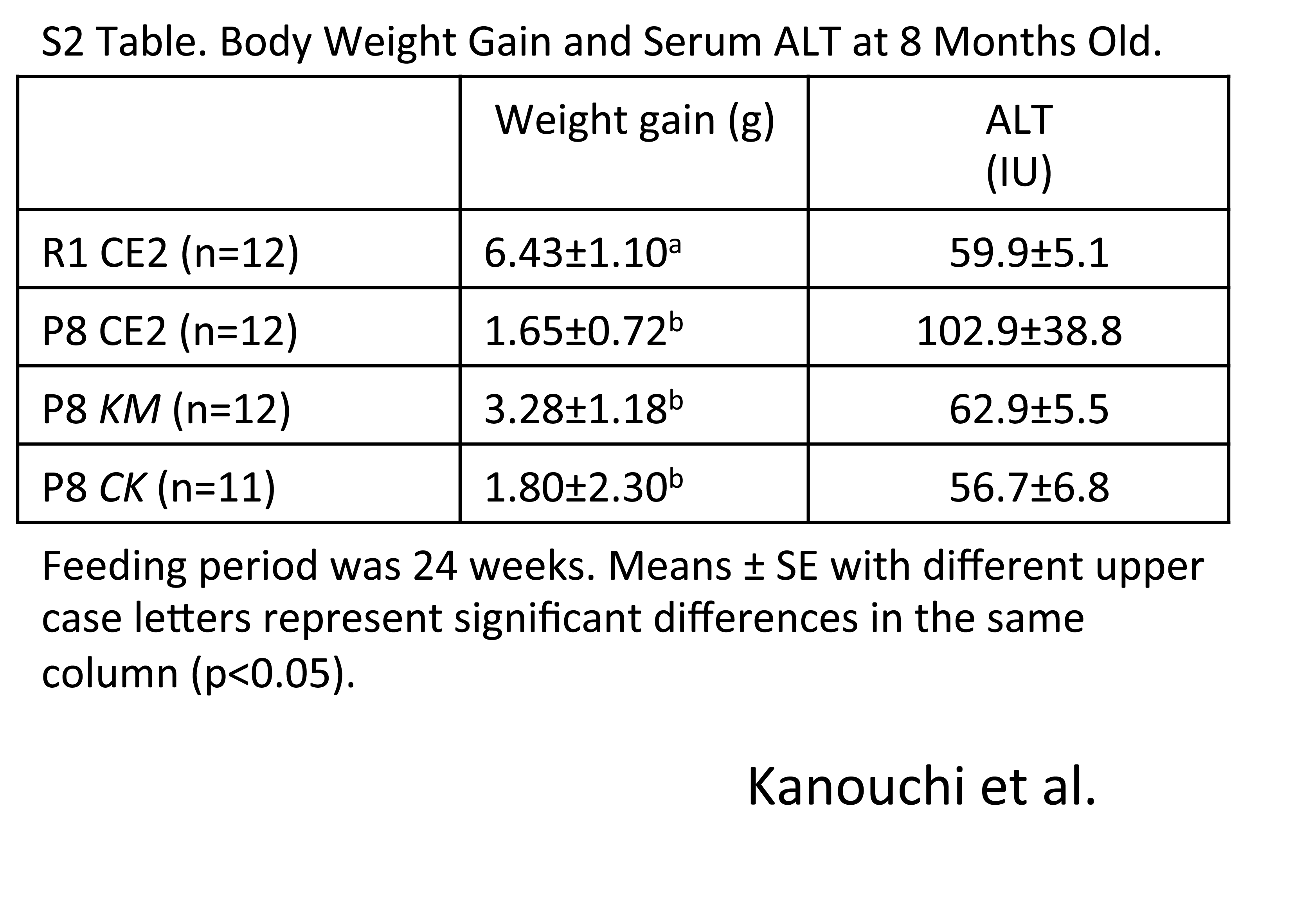

Supplement: S2 Table — (TIF) [file pone.0150796.s002.tif]

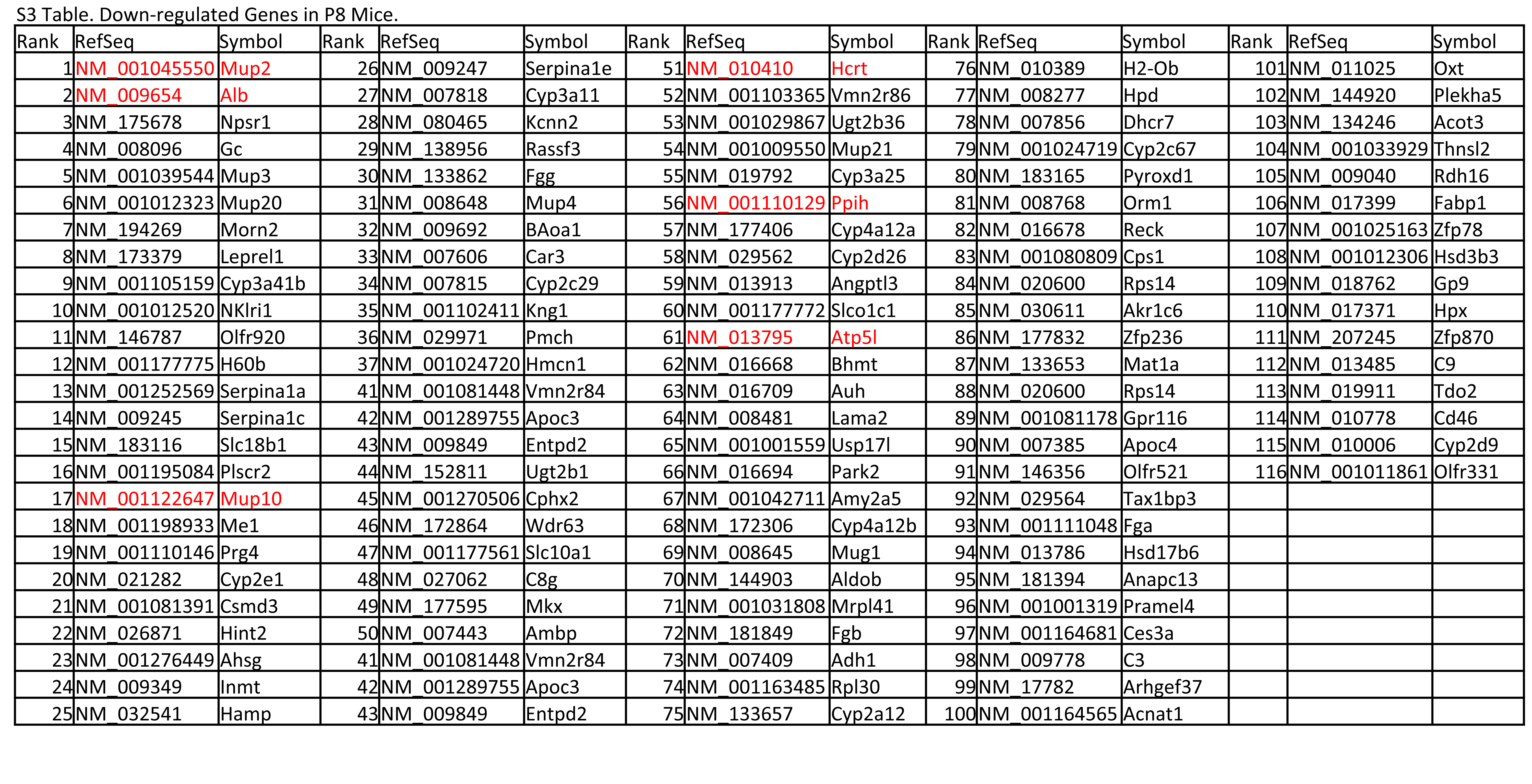

Supplement: S3 Table — (TIF) [file pone.0150796.s003.tif]

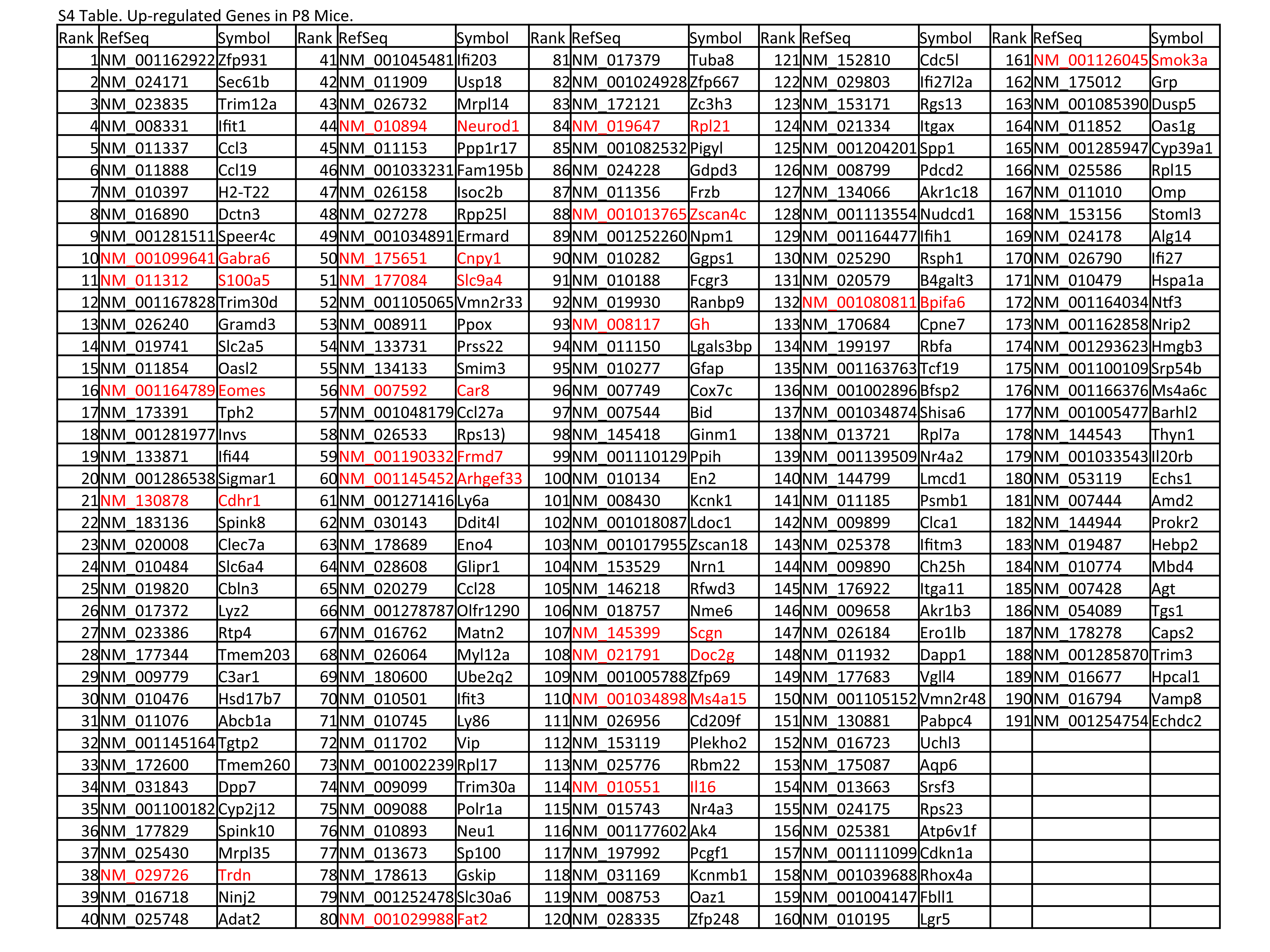

Supplement: S4 Table — (TIF) [file pone.0150796.s004.tif]
